# Endothelial‐Targeted Metallothionein‐2 shRNA Nanoparticles Alleviate Migraine‐Like Symptoms

**DOI:** 10.1111/cns.70643

**Published:** 2025-11-05

**Authors:** Chenlu Zhu, Xiao Ren, Zongxing He, Jinggui Gao, Kaibo Zhang, Tianxiao Wang, Cheng Peng, Jianfeng Li, Jisong Guan, Yonggang Wang

**Affiliations:** ^1^ Headache Center, Department of Neurology, Beijing Tiantan Hospital Capital Medical University Beijing China; ^2^ Department of Neurology The First Affiliated Hospital of Shandong First Medical University & Shandong Provincial Qianfoshan Hospital Jinan China; ^3^ School of Life Science and Technology ShanghaiTech University Shanghai China; ^4^ Department of Neurology, the First Affiliated Hospital, Jiangxi Medical College Nanchang University Nanchang China; ^5^ Postdoctoral Research Station, the First Affiliated Hospital, Jiangxi Medical College Nanchang University Nanchang China; ^6^ Department of Neurology The First Affiliated Hospital of Zhengzhou University Henan China; ^7^ Department of Neurology Lanzhou University Second Hospital Lanzhou China

## Abstract

**Aim:**

Migraine is a common chronic neurological disorder manifesting as recurrent moderate‐to‐severe headaches. It continues to present therapeutic challenges due to population heterogeneity in treatment response. This study investigates the unexplored role of metallothionein‐2 (Mt2) in migraine pathophysiology.

**Methods:**

We employed the EGR1‐GFP reporter system to identify activated cortical cells induced by nitroglycerin (NTG). RNA sequencing of somatosensory activated cortex cells revealed marked Mt2 upregulation particularly in vascular endothelial cells. To elucidate Mt2's function in migraine pathogenesis, endothelial‐targeted nanoparticles encapsulating Mt2‐shRNA were engineered and administered via tail‐vein injection in migraine model mice. Behavioral assays assessed photophobia and hyperalgesia, while two‐photon microscopy evaluated cerebral vasodilation. Pathway enrichment analysis identified critical biological pathways linked to Mt2 activity.

**Results:**

Suppression of Mt2 in endothelial cells via shRNA nanoparticles alleviated migraine‐related behaviors, including photophobia and hyperalgesia. RNA‐seq and pathway analysis highlighted Mt2's involvement in cerebrovascular endothelial cell development, migration, and inflammation pathways. Crucially, two‐photon imaging demonstrated that downregulation of Mt2 markedly attenuated NTG‐induced cerebral vasodilation.

**Conclusion:**

This study establishes Mt2 as a regulator of vascular tone and inflammatory signaling in migraine pathogenesis, proposing novel therapeutic targets. These findings provide insight in understanding the interplay between vascular dysfunction and migraine.

## Graphical abstract

This schematic illustrates the therapeutic effects of endothelial‐targeted Mt2‐shRNA nanoparticles in a migraine model. In saline‐treated controls (Normal), cerebral vasculature remains balanced. Nitroglycerin (NTG) triggers pathological vasodilation in the migraine mice model, accompanied by elevated Mt2, nitric oxide (NO), and pro‐inflammatory markers (CGRP, IL‐6, TNF‐α), alongside neuronal activation. Administration of Mt2‐shRNA nanoparticles reverses these effects: Mt2 suppression reduces NO production via NOS2 downregulation, normalizes vascular tone, and attenuates neuroinflammation. Concurrently, activated neurons decrease, demonstrating dual modulation of cerebrovascular dysfunction and inflammatory signaling. Two‐photon imaging validates restored vascular dynamics, highlighting Mt2 as a pivotal therapeutic target for migraine intervention.

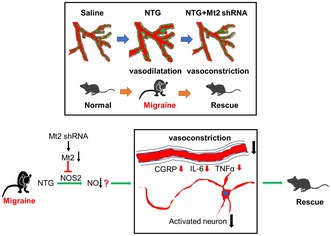

**Abbreviations**
CGRP: calcitonin gene‐related peptide
CM: chronic migraine
DEGs: differentially expressed genes
Mt2: metallothionein‐2
NTG: nitroglycerin
PACAP: pituitary adenylyl cyclase‐activating polypeptide
VIP: vasoactive intestinal peptide

## 1 Background

Chronic migraine (CM), a primary functional brain disease characterized by unilateral, throbbing, and intense headaches, often accompanied by photophobia and phonophobia [1]. This condition exhibits marked female predominance, affecting women at approximately twice the incidence rate observed in males [2, 3]. Despite its substantial socioeconomic impact through workforce productivity loss and diminished quality of life, therapeutic development remains constrained by incomplete understanding of its pathophysiological mechanisms [4, 5, 6].

Emerging evidence implicates neurovascular dysregulation in migraine pathogenesis, particularly through abnormal perivascular inflammatory mediator release. Key molecular players include calcitonin gene‐related peptide (CGRP), pituitary adenylyl cyclase‐activating polypeptide (PACAP), vasoactive intestinal peptide (VIP), interleukin‐6 (IL‐6), and tumor necrosis factor‐alpha (TNF‐α) [7, 8, 9]. These inflammatory substances are released following activation of the trigeminovascular pathway, which facilitates central sensitization and nociceptive transmission to cortical regions—particularly the somatosensory cortex—through aberrant neuronal and glial activation in the central nervous system [10]. However, the precise molecular cascades governing this neuroinflammatory axis remain poorly delineated.

Recent investigations have identified metallothionein‐2 (Mt2), a cysteine‐rich metalloprotein involved in metal ion homeostasis, as a novel modulator of pain pathophysiology. Beyond its canonical detoxification functions, Mt2 demonstrates regulatory capacity in both neuropathic and inflammatory pain states through endothelial‐mediated inflammatory mediator release. Mechanistically, Mt2 is reported to interact with nuclear factor‐kappa B (NF‐κB) signaling as a master regulator of pro‐inflammatory cytokine production implicated in central sensitization processes [11]. This intersection between metalloprotein function and pain neurobiology suggests Mt2 may constitute a previously unrecognized node in migraine‐related pathophysiology.

We propose a novel hypothesis that Mt2 modulates CM by regulating cerebral endothelial inflammatory mediator secretion, and neurovascular tone. This study aims to investigate Mt2's potential as a therapeutic target while elucidating its specific molecular mechanisms in migraine pathophysiology.

## 2 Methods

### 2.1 Animals

Male C57BL/6 mice (20–25 g) and EGR1‐EGFP transgenic mice were utilized in this study. The selection of male mice was based on their relatively stable hormonal profiles compared to females, which minimizes variability in neurobehavioral responses. All animals were procured from Shanghai University of Science and Technology (Shanghai, China) or generated in‐house (EGR1‐EGFP line). Mice were maintained under standardized conditions, including a 12‐h light–dark cycle, controlled temperature and humidity, with ad libitum access to food and water. All experimental protocols were approved by the Institutional Animal Care and Use Committee (IACUC) (Approval No. 20240827003) and conducted in compliance with NIH guidelines (Guide for the Care and Use of Laboratory Animals, 8th edition).

### 2.2 CM Model Induction

Migraine pathophysiology is characterized by cerebral vasodilation and trigeminovascular system activation. Notably, nitroglycerin (NTG) administration is a recognized pharmacological trigger for migraine attacks in clinical populations, primarily through nitric oxide (NO)‐mediated vasodilation and subsequent neuroinflammatory cascades. Building on this mechanism, a validated CM‐like model was established through repeated intraperitoneal (ip) administration of NTG (5 mg/mL stock solution containing 30% ethanol, 30% propylene glycol, and water; Beijing Yimin Pharmaceutical Co.). The NTG working solution (1 mg/mL) was prepared by fivefold dilution in sterile saline immediately before use. Mice received intraperitoneal injections (10 mg/kg) on Days 1, 3, 5, 7, and 9. Control animals received equivalent volumes of saline following the same schedule. All injections were performed under dim light to prevent NTG photodegradation.

### 2.3 Intravenous Delivery of Mt2 shRNA by Lipid Nanoparticles

To deliver Mt2 shRNA to cerebral blood vessels, based on previous publications, biodistribution analysis via immunofluorescence confirmed selective nanoparticle accumulation in cerebrovascular endothelial cells [12].

First, three Mt2‐targeting shRNA sequencings were designed using the siRNA Selection Program (Whitehead Institute) and cloned into PLKO.1 lentiviral vectors. HEK293T cells overexpressing Myc‐tagged Mt2 (pcDNA3.1‐Mt2‐Myc) were transduced with individual shRNAs. qRT‐PCR and western blot identified shMt2‐2 as the most effective.

Second, the validated shMt2‐2 sequence was encapsulated in poly (β‐amino ester) (PBAE) nanoparticles (11C10E1 polymer, *n* = 20.58) using a solvent displacement method [13]. Nanoparticles (0.45 mg/kg) were administered via tail vein injection 24 h prior to each NTG/saline treatment. Four experimental cohorts were established: saline group: received saline injections (*n* = 10); NTG group: NTG‐treated mice (*n* = 12); NTG + Mt2‐shRNA group: NTG‐treated mice with nanoparticle‐mediated Mt2 knockdown (*n* = 12); NTG + Ctrl‐shRNA group: NTG‐treated mice receiving scrambled shRNA nanoparticles (*n* = 12).

### 2.4 Behavioral Test

All behavioral experiments were conducted between 9:00 a.m. and 3:00 p.m. Mice were habituated to the testing environment for 30 min pre‐assessment. All equipment was cleaned with 75% alcohol after each test to exclude the effect of odors and feces. Behavioral assessments were performed both 2 h before and after drug interventions to evaluate their effects. For the mechanical allodynia test, von Frey filaments (0.008–4 g force range) were applied perpendicularly to the periorbital region and hind paw plantar surfaces. A modified up‐down method was employed, with positive responses (rapid withdrawal, head shaking, or paw flicking) recorded as “X” and negative responses as “O.” The 50% withdrawal threshold was calculated using the online von Frey calculator (https://bioapps.shinyapps.io/von_frey_app/), as previously described [14]. The hot plate test is employed in migraine research to evaluate thermal hyperalgesia, a hallmark of central sensitization observed in migraine pathophysiology. As previously described, mice were placed on a 55°C ± 0.5°C hot plate (Ugo Basile) [15]. Latency to hind paw withdrawal/licking was recorded (cutoff: 30 s). Two trials were averaged with a 15‐min inter‐trial interval. Clinical evidence demonstrates that photophobia—a heightened sensitivity to light—is a hallmark symptom in migraine patients during attacks. To recapitulate this clinical phenotype in our preclinical model, the light‐aversive test was conducted. The light/dark box transitions (300 lx vs. 0 lx chambers) were monitored for 10 min using ANY‐maze software. Light aversion was quantified as time spent in the illuminated chamber.

### 2.5 Western Blotting Analysis

Mice were transcranial perfused with ice‐cold PBS 12 h post‐final intervention. As previously described, brain tissues were homogenized in RIPA buffer containing protease inhibitors to harvest the protein lysates [16]. Protein concentration was quantified by BCA Protein Assay Kit [17, 18]. Protein lysates (30 μg/lane) were resolved on 12% SDS‐PAGE gels and transferred to PVDF membranes. After blocking with 5% non‐fat milk (30 min) and washed three times in PBS consequently, membranes were probed with antibodies including rabbit anti‐Mt2 (1:500; ABclonal, A2018), mouse anti‐Myc (1:500; Abcam, ab32), rabbit anti‐GAPDH (1:5000; CST, 2118S). Finally, HRP‐conjugated secondary antibodies (1:10,000; Bioworld/Jackson ImmunoResearch) were detected via ECL (Bio‐Rad). Band intensities were quantified using ImageJ (NIH) with GAPDH normalization.

### 2.6 Quantitative Real‐Time PCR

Total RNA was isolated from brain tissues of experimental mice using TRIzol reagent (Invitrogen, 15596026) and reverse‐transcribed into cDNA using the TransScript First‐Strand cDNA Synthesis SuperMix (TransGen Biotech, AT301‐02). Quantitative PCR amplification was performed on a QuantStudio 6 Flex system (Applied Biosystems) with 2 × SYBR Green Master Mix (Bimake, B21203) under optimized cycling conditions: 95°C for 20 s (initial denaturation), followed by 40 cycles of 95°C for 15 s, 60°C for 30 s, and 72°C for 30 s. Gene primers (Table S1) were designed using Primer‐BLAST (NCBI) with amplicon lengths ranging 80–150 bp. Melt curve analysis confirmed primer specificity. Relative mRNA expression was normalized to GAPDH using the 2^−ΔΔCt^ method.

### 2.7 Immunofluorescence Staining

Mice were anesthetized by 3% isoflurane and perfused with phosphate‐buffered saline (PBS, 0.01 M, pH = 7.4), then followed by 4% paraformaldehyde (PFA), and brains were post‐fix overnight with 4% PFA. Next, brains were cryoprotected in 20% and 30% sucrose gradients (24 h each) and sectioned coronally at 40 μm thickness using a Leica VT1200S vibratome [19]. Free‐floating sections were permeabilized with 0.3% Triton X‐100 (Sigma, T8787) in PBS, blocked with 5% normal donkey serum (Jackson ImmunoResearch, 017‐000‐121), and incubated with primary antibodies (4°C, 48 h) including anti‐Mt2 (ABclonal, A2018; 1:500), anti‐CD31 (Thermo Fisher, 14‐0311‐82; 1:500), anti‐NeuN (Abcam, ab104224; 1:500), anti‐GFAP (Abcam, ab4648; 1:500), anti‐Iba1 (Abcam, ab289874; 1:500), anti‐cFos (Abcam, ab222699; 1:500). After PBS washes, sections were incubated with Alexa Fluor‐conjugated secondary antibodies (1:1000; Thermo Fisher) for 2 h at 25°C. Nuclei were counterstained with DAPI (Sigma, D9542; 1 μg/mL). Z‐stack images (1 μm intervals) were acquired using a Nikon A1R confocal microscope and analyzed with NIS‐Elements AR 5.21 software. Co‐localization was quantified using Pearson's correlation coefficient (ImageJ JACoP plugin).

### 2.8 Surgery and In Vivo Two‐Photon Imaging

To facilitate the observation of intracranial vasodilation and constriction using two‐photon imaging technology, a cranial window (3 mm diameter) was surgically implanted over the somatosensory cortex (coordinates: AP −1.8 mm, ML 2.5 mm from bregma) under isoflurane anesthesia (1.5%–2%). After 4‐week recovery, mice received intravenous Texas Red‐dextran (70 kDa; Thermo Fisher, D1864; 5 mg/mL) to label vasculature. Time‐lapse imaging was performed using an Olympus FV1200MPE multiphoton microscope (excitation: 1100 nm; emission: 575–630 nm). Vascular diameters were measured at baseline and 2 h post‐NTG injection (day 9) using ImageJ. The percentage change in diameter is calculated manually based on the above data.

### 2.9 Histiocyte Flow Cytometry Sorting and Bulk RNA Sequencing

For the activity‐dependent transcriptomics in EGR1‐EGFP mice, somatosensory cortex tissues from NTG‐treated and saline‐control EGR1‐EGFP mice (*n* = 3/group, male, 8–10 weeks) were dissociated using the Adult Brain Dissociation Kit (Miltenyi, 130‐107‐677). EGR1‐activated cells were sorted via FACS (BD FACSAria III) with GFP excitation/emission (488/530 nm). For the endothelial‐specific transcriptomics, CD31‐positive endothelial cells were isolated from the somatosensory cortex of Mt2 shRNA group and Mt2 ctrl shRNA group mice (*n* = 3/group) using anti‐CD31‐PE/Cy7 (BioLegend, 102418; 1:200) staining.

For the isolated cells, we extracted total RNA and assessed its integrity using the Agilent Bioanalyzer 2100 (Agilent Technologies, Santa Clara, CA, US). RNA samples that met quality standards were further purified with the RNeasy micro kit (Cat#74004, QIAGEN) and RNase‐Free DNase Set (Cat#79254, QIAGEN). After quality checks, the sequencing reads were trimmed and low‐quality sequences were filtered before being mapped to the mouse reference genome (GRCm38) using HISAT (version 2.0.4).

Gene expression levels were quantified using Stringtie to count the detected genes, and the data were normalized to calculate the FPKM (fragments per kilobase per million mapped fragments) values for the exon model. HISAT was used for mapping, while HTSeq and differentially expressed genes (DEGs) sequencing were employed for gene expression quantification and data normalization, respectively. DEGs were identified as those with an adjusted *p* value ≤ 0.05 and a Log2(fold‐change) of > 1 or < −1, ensuring technical robustness and biological relevance.

### 2.10 Statistical Analysis

Data are presented as mean ± standard error of the mean (SEM). All statistical analyses were conducted using PRISM 8 software and SPSS 22.0. We assessed whether the data followed a normal distribution using the Kolmogorov–Smirnov test method, which confirmed they follow a normal distribution. We employed two‐sided unpaired Student's *t*‐tests and Tukey's post hoc tests to determine the statistical significance between two different groups. For data involving more than two groups, we utilized one‐way analysis of variance (ANOVA) followed by post hoc tests to identify statistical significance. In the case of behavioral experiments, we applied two‐way ANOVA with Tukey's post hoc test for analysis. All experiments adhered to ARRIVE guidelines, with investigators blinded to treatment groups during data acquisition and analysis. Statistical significance was set at *p* < 0.05.

## 3 Results

### 3.1 NTG Induces Migraine‐Like Behaviors and Upregulates Mt2 Expression

**FIGURE 1:**
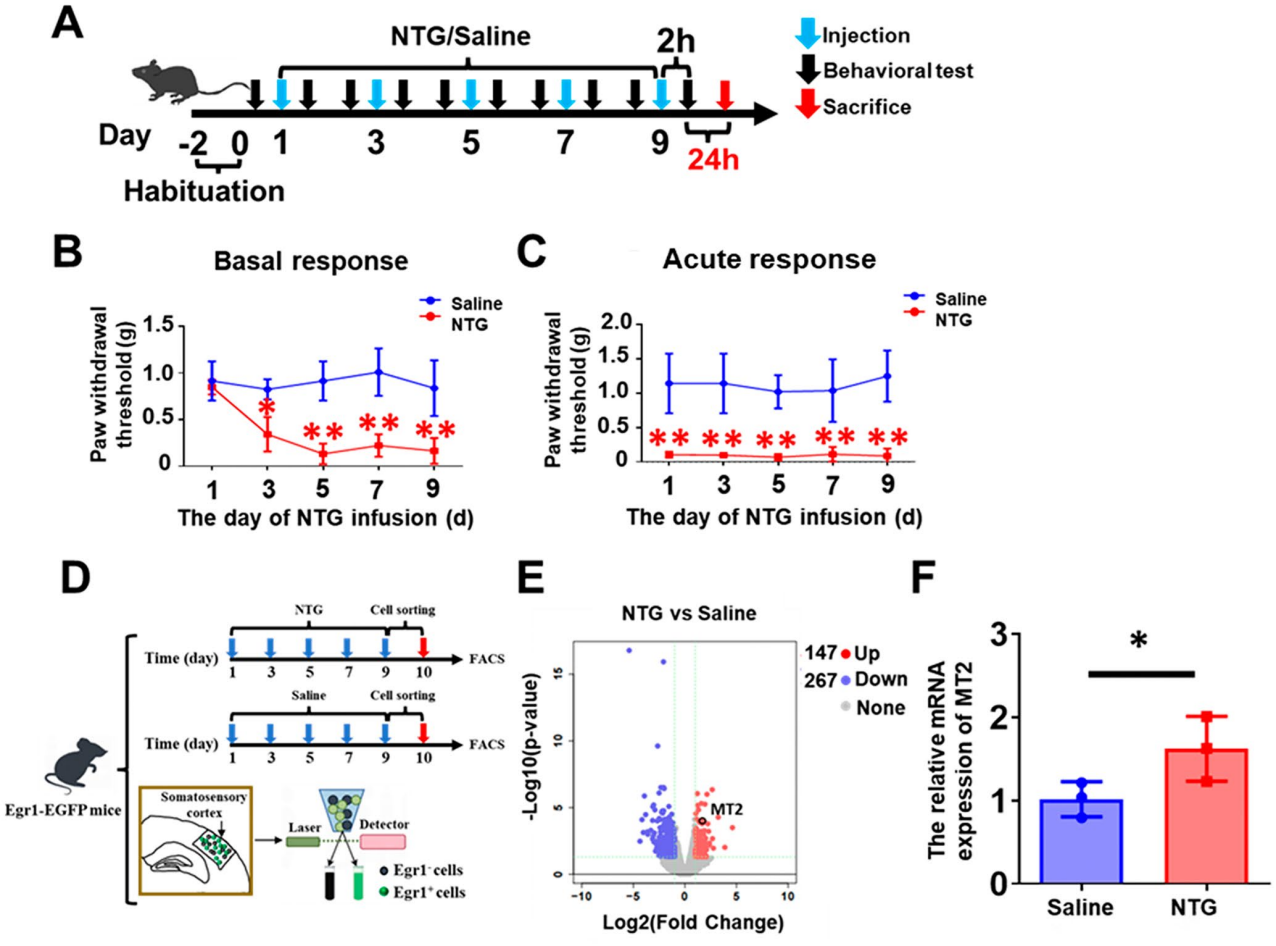
NTG induces migraine‐like behaviors and upregulates Mt2 expression. (A) Representative schematic diagrams for drug administration and behavioral tests. (B, C) Repeated NTG administration induced mechanical hyperalgesia including basal responses (B) and acute responses (C). Two‐way ANOVA with the Tukey post hoc tests, *p < 0.05, **p < 0.01, *n* = 8/group. (D) Flowchart of EGR1‐positive cell acquisition in migraine mouse. (E) RNA sequencing data based on egr1‐positive cells of somatosensory cortex in EGR1‐EGFP mice between the NTG group and the saline group. The NTG group compared with the saline group, *n* = 3/group. (F) The relative Mt2 mRNA expression level increases in the NTG group when compared with the saline group. One‐way ANOVA with the Tukey post hoc tests, *p < 0.05, **p < 0.01, *n* = 3/group.

NTG, a nitric oxide (NO) donor, was selected to establish the migraine model due to its well‐documented capacity to trigger migraine‐like attacks in humans through NO‐mediated trigeminovascular activation and neuroinflammatory cascades [20]. To recapitulate CM pathophysiology, we administered NTG (10 mg/kg, ip) every other day for 9 days, a regimen standardized by Burgos‐Vega et al. to mimic CM progression through sustained central sensitization and vascular dysregulation [21]. To validate this model, we first habituated mice for 2 days to minimize stress‐induced variability. Then, the EGR1‐EGFP mice received intraperitoneal injections of NTG or the same dose of saline (control) as illustrated in Figure 1A. Behavioral assessments were conducted 2 h before (basal response) and 2 h after (acute response) injection. The paw withdrawal threshold of the NTG group was significantly reduced in both basal and acute responses (Figure 1B,C), corresponding with previously reported [21]. It thus interests us to ask which brain region responds quickly to migraine‐like pain signals. As our previously published data [22], state, NTG‐induced migraine mice exhibited significantly increased neuronal activities in the somatosensory cortex by recording EGR1 expression. Besides, the somatosensory cortex also plays a critical role in processing cephalic nociception and mediating migraine‐related allodynia as indicated in clinical and animal studies [22]. Thus, we focus on how the somatosensory cortex changes in migraine pathology. Transcriptomic profiling of EGR1^+^ neurons isolated from the somatosensory cortex as shown in Figure 1D, identified 412 DEGs including 147 upregulated genes and 267 downregulated genes. We selected the top downregulated and upregulated genes for further analysis. We were surprised to find that Mt2 expression levels were significantly increased (Figure 1E,F). These data support Philippa Pettingill's view that mutations of TRESK result in the overexpression of Mt2, enhancing trigeminal ganglion excitability in migraine [23]. It is interested to ask what the function of Mt2 is in migraine pathology.

### 3.2 Mt2 Exhibits Vascular Endothelial and Microglia‐Specific Expression Patterns

In the central nervous system, previous studies have localized the expression of Mt2 to vascular endothelial cells and neurons in the spinal dorsal horn during neuropathic pain [11]. Interestingly, our immunofluorescence co‐staining revealed distinct expression patterns in the somatosensory cortex of the migraine model. In our data, we found that Mt2 expression was predominantly co‐localized with CD31 (a marker for endothelial cells) (Figure S1A) and Iba1 (a marker for microglia) (Figure S1B), but little to no co‐localization with NeuN (a marker for mature neurons) (Figure S1C). Recent studies highlight the pivotal regulatory role of vascular endothelial cells in the pathophysiology of migraine. These cells contribute to neurovascular coupling abnormalities and cortical spreading depression (CSD) by modulating vascular tone, releasing inflammatory mediators, and regulating blood–brain barrier permeability. Notably, the aberrant overexpression of Mt2 in the vascular endothelium may provide novel insights into migraine mechanisms.

### 3.3 Mt2 Endothelial‐Targeted Nanoparticles Construction

**FIGURE 2:**
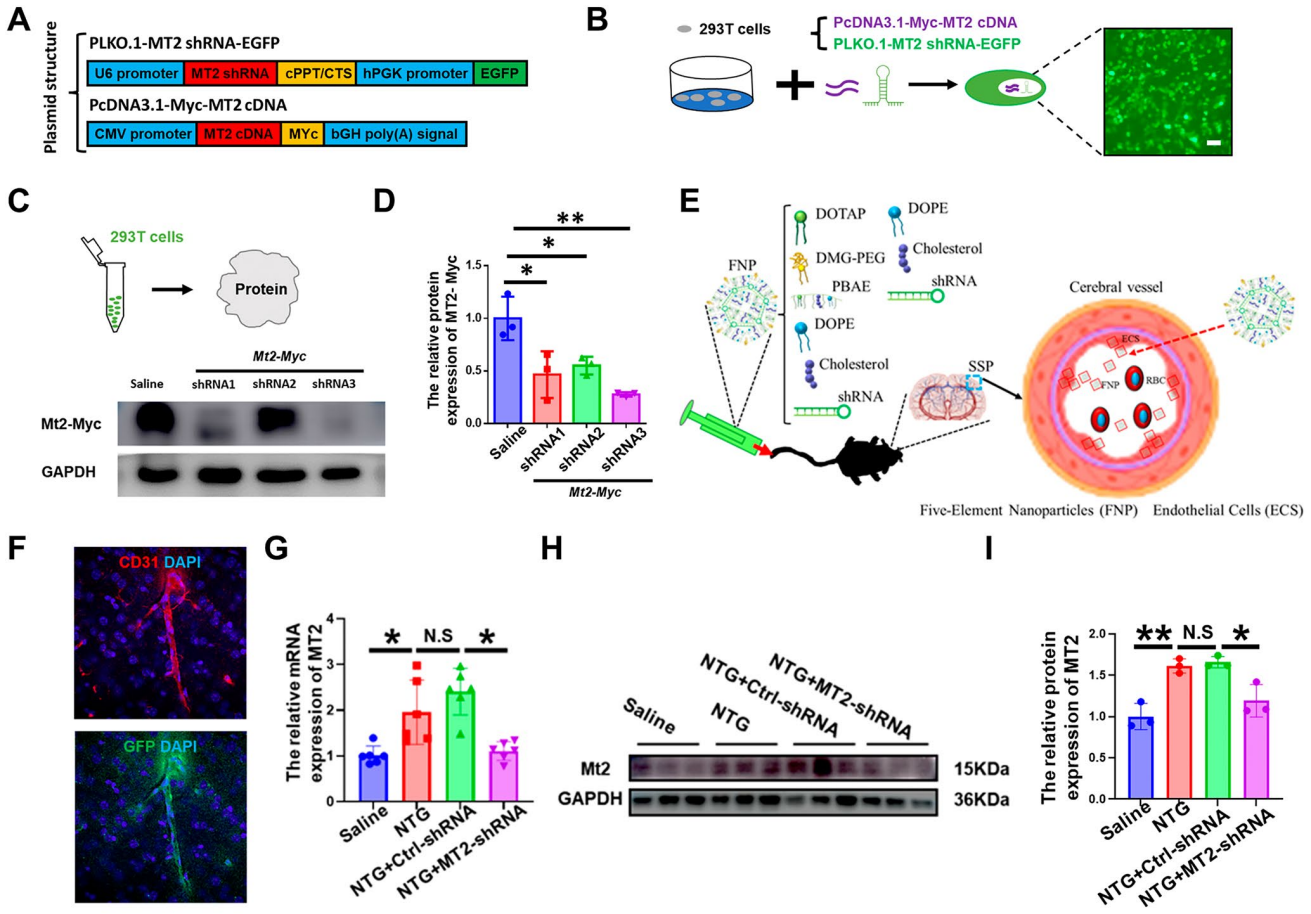
Mt2 endothelial‐targeted nanoparticles construction. (A) Plasmid structure model diagram including PLKO.1‐Mt2 shRNA‐EGFP and PcDNA3.1‐Myc‐Mt2 cDNA. (B) T cells successful transfection was confirmed via fluorescence microscopy, which detected robust green fluorescent protein (GFP) expression in HEK‐293 cells 48 h post‐lipofection with the recombinant plasmid (C). The transfected 293 T cells were collected, and the knockdown effect of Mt2 gene was detected by WB. (D) The Mt2 gene was knocked down. *t*‐test, *p < 0.05, **p < 0.01, *n* = 3/group. (E) Schematic illustration of five‐element nanoparticles (FNPs) and its injection into mouse brain vessel endothelial cells (ECS) via caudal vein. (F) Representative images of CD31 (red) and GFP (green) immunofluorescence labeling in the somatosensory cortex. Scale bars =10 μm. (G–I) Mt2 gene was knocked down by Mt2‐shRNA in mRNA level (G) and protein level (I). *t*‐test, *p < 0.05, **p < 0.01, *n* = 3–6/group.

It is interesting to ask what is the potential function of vascular Mt2 in migraine pathology. First, we designed three Mt2‐specific shRNA sequences using the siDESIGN Center, cloned into PLKO.1‐puro lentiviral vectors as shown in Figure 2A,B. Western blot analysis demonstrated shRNA‐3 has the most effective suppression level by calculating Myc expression (Figure 2C,D). Second, we selected shRNA‐3 and packaged it into engineered endothelial‐targeted nanoparticles for in vivo delivery (Figure 2E). Immunofluorescence analysis using CD31 (a marker for endothelial cells) and GFP‐labeled Mt2 nanoparticles revealed predominant colocalization of Mt2 nanoparticles with vascular endothelial cells, confirming the nanoparticles' high specificity to vascular tissue (Figure 2F). To further validate Mt2‐shRNA nanoparticles' function in vivo, mice were separated into four groups, including saline, NTG, NTG + Ctrl‐shRNA, and NTG + Mt2‐shRNA. The western blot analysis indicated the NTG group significantly increased the expression of MT2 in endothelial cells compared with the saline group, but significantly downregulated in the NTG + Mt2‐shRNA group (Figure 2I). Taken together, these data validate the specificity of the Mt2 shRNA nanoparticle delivery system in endothelial cells.

### 3.4 Mt2 Knockdown Attenuates NTG‐Induced Migraine‐Like Behaviors

**FIGURE 3:**
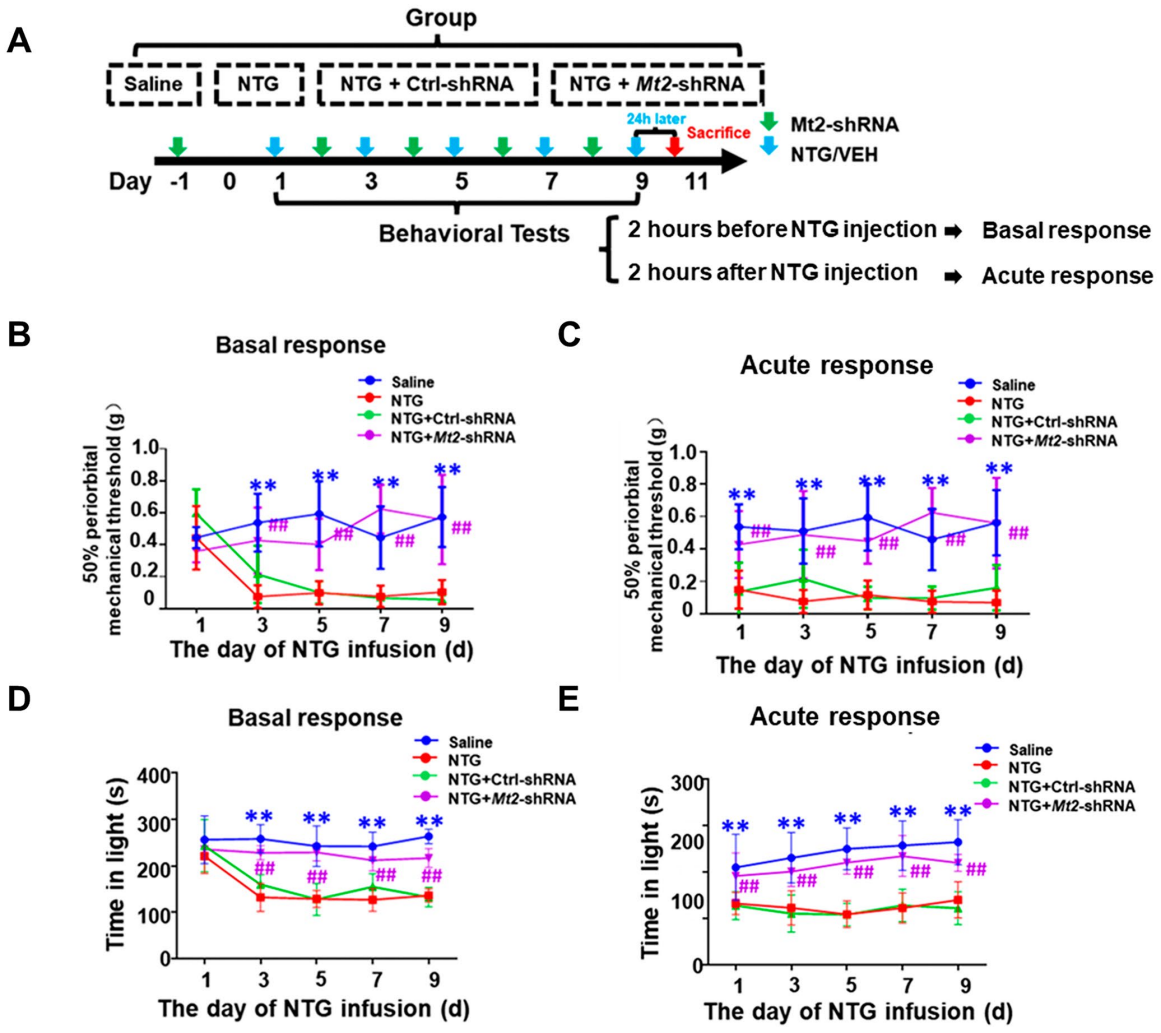
Mt2 knockdown attenuates NTG‐induced migraine‐like behaviors. (A) Representative schematic diagrams and procedures for drug administration and behavioral tests. (B, C) Repeated Mt2 shRNA administration decreased periorbital mechanical hyperalgesia induced by NTG including chronic responses (B) and acute responses (C). Two‐way ANOVA with the Tukey post hoc tests, ##*p* < 0.01, NTG + Mt2 shRNA group compared with NTG + Ctrl shRNA group, *n* = 8/group, **p < 0.01, NTG group compared with saline group, *n* = 8/group. (D, E). Repeated Mt2 shRNA administration decreased time in light by NTG‐induced photophobia including basal responses (D) and acute responses (E) in light and dark test. Two‐way ANOVA with the Tukey post hoc tests, ##*p* < 0.01, NTG + Mt2 shRNA group compared with NTG + Ctrl shRNA group, *n* = 8/group, **p < 0.01, NTG group compared with saline group, *n* = 8/group.

It is interesting to know whether knockdown of the expression of Mt2 in endothelial cells could attenuate NTG‐induced migraine‐like behaviors. The timeline indicates the days of treatment, with behavioral tests conducted on specific days (Days 1, 3, 5, 7, 9). Twenty‐four hours after the last treatment on Day 10, mice were sacrificed. The behavioral tests include the periorbital mechanical threshold test, light aversion test and paw mechanical pain threshold test (Figure 3A). In the periorbital mechanical threshold test, the red line (NTG group) indicates a significant decrease in periorbital mechanical threshold. The green line (NTG + Ctr‐shRNA group) is similar to the NTG group, while the purple line (NTG + Mt2‐shRNA group) shows a different pattern, significantly attenuating the basal and acute periorbital mechanical threshold (Figure 3B,C). These data were further supported by the paw mechanical pain threshold test (Figure S2A–C). In order to further validate its function on migraine‐like behavior, we performed the light aversion test, which also revealed that downregulating the expression of Mt2 in endothelial cells could alleviate the photophobia phenotype (Figure 3D,E). Taken together, these findings support the view that endothelial Mt2 knockdown alleviates migraine‐like behaviors, offering a potential therapeutic strategy for migraine.

### 3.5 Endothelial Mt2 Regulates Neuronal Activation via Vascular–Neural Crosstalk

**FIGURE 4:**
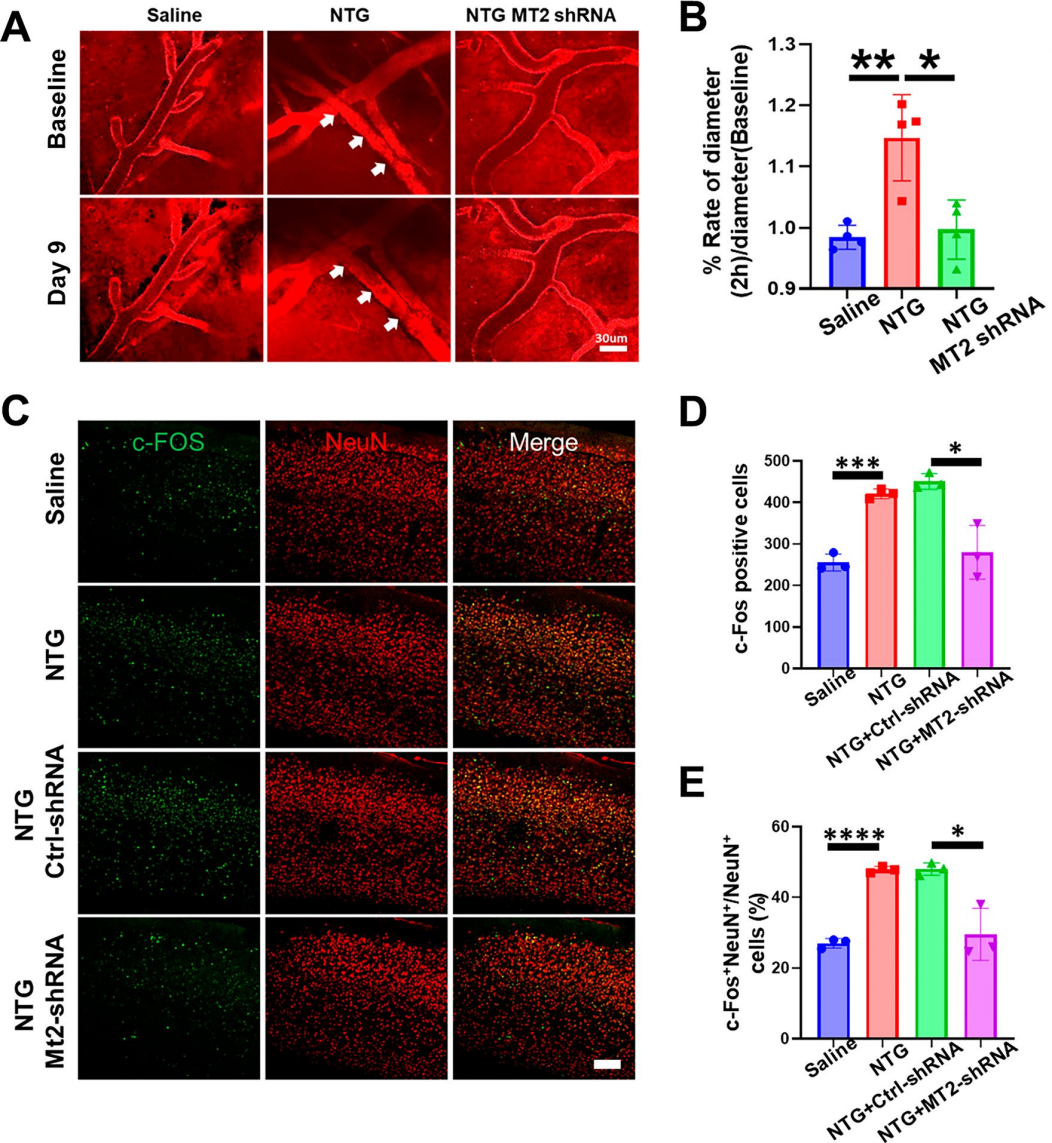
Endothelial Mt2 regulates neuronal activation via vascular–neural crosstalk. (A) Representative images of vessel in mouse brain captured by two‐photon confocal microscopy. Scar bar 30 μm, *n* = 3/group. (B) Statistical analysis of vessel diameter alteration and showed that Mt2 shRNA decreased the vessel dilation induced by NTG including % rate of diameter (2 h)/diameter (baseline). *t*‐test, *p < 0.05, **p < 0.01, *n* = 4/group. (C) Representative pictures of c‐Fos (green) and NeuN (red) immunofluorescence labeling in the somatosensory cortex. Scale bars =100 μm. (D) Statistical analysis of the c‐Fos‐positive cells in the groups. *t*‐test, *p < 0.05, ***p < 0.001, *n* = 3/group. (E) Statistical analysis of c‐Fos^+^NeuN^+^/NeuN^+^ cells (%) in the groups. *t*‐test, *p < 0.05, ****p < 0.0001, *n* = 3/group.

It is interesting to explore how endothelial Mt2 regulates migraine‐like behaviors. Endothelial cells critically regulate cerebrovascular tone and blood–brain barrier integrity, forming the structural basis of neurovascular networks that mediate bidirectional communication between the vasculature and neural circuits. Through dynamic secretion of vasoactive mediators (e.g., NO, CGRP) and inflammatory cytokines, these cells modulate neuronal excitability via perivascular neurotransmitter gradients and neuroinflammation, ultimately contributing to migraine pathogenesis through amplified trigeminovascular signaling. It is widely accepted the view that the NTG induces vasodilation that triggers migraine‐like pain. To test this view, we conducted in vivo cerebrovascular imaging using two‐photon microscopy to monitor real‐time vascular dynamics in the brains of awake behaving mice. NTG‐treated mice revealed pronounced cerebrovascular dilation. However, by extraneously using Mt2‐shRNA nanoparticles, we found the vasodilation phenotype was dramatically attenuated (Figure 4A,B). Considering the close relationship between abnormal vasodilation and neuronal abnormal activation, we co‐stained the c‐FOS (a marker for activated neurons) and NEUN (a marker for mature neurons); we surprisingly found the abnormally activated neurons in the NTG group were significantly reduced by quantifying the c‐Fos cell density and the c‐Fos^+^NeuN^+^ cells (Figure 4C–E). These data provide evidence that Mt2 may regulate the vasoconstriction and vasodilation involved in the pathogenesis of migraine.

### 3.6 Molecular Pathway Underlying Endothelial Mt2‐Mediated Migraine Regulation

**FIGURE 5:**
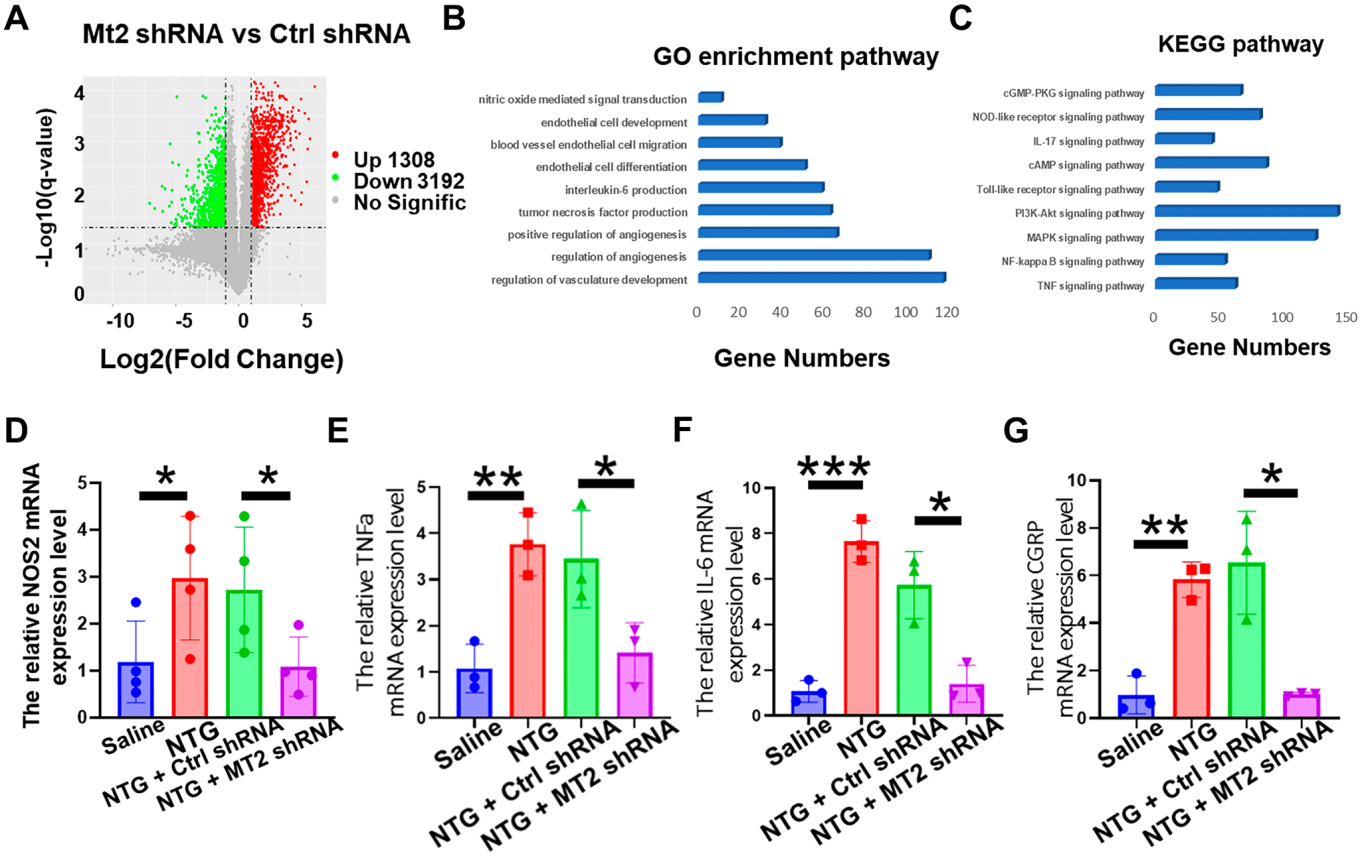
Molecular pathway underlying endothelial Mt2‐mediated migraine regulation. (A) RNA sequencing data between the Mt2 shRNA group and Ctrl shRNA group. (B) GO enrichment analysis based on RNA sequencing data. (C) KEGG pathway analysis. (D–G) Based on the sequencing results, it was found through qPCR test that Mt2shNRA promoted the decreased expression of blood vessel and inflammation‐related molecules including NOS2 (D), TNF‐α (E), IL‐6 (F), CGRP G induced by NTG. *t*‐test, *p < 0.05, **p < 0.01, ***p < 0.001, *n* = 3/group.

Endothelia cell Mt2 was critical in the migraine pathology; it is of interest to explore the molecular pathway involved. Transcriptomic profiling of endothelial cells following MT2 knockdown revealed substantial transcriptomic reprogramming, with 1308 genes significantly upregulated and 3192 genes downregulated (Figure 5A). Systematic analysis of these DEGs through gene ontology (GO) enrichment demonstrated their strong association with critical biological processes including: (1) vascular morphogenesis and angiogenesis, (2) cytokine secretion dynamics (particularly IL‐6 and TNF‐α), (3) endothelial cell differentiation and motility, and (4) nitric oxide‐mediated signaling transduction (Figure 5B). Complementary KEGG pathway analysis further established Mt2's central role in inflammatory regulation, with pronounced enrichment in three key signaling cascades: the PI3K‐Akt survival pathway, MAPK‐mediated inflammatory response, and cAMP‐dependent neurotransmission pathways (Figure 5C).

The enrichment analysis revealed that Mt2 downregulation is highly associated with the inflammatory‐related pathway. We conducted qPCR validation of RNA sequencing results of top downregulated inflammatory genes. This analysis revealed significant downregulation of NOS2 (inducible nitric oxide synthase) along with key migraine‐associated inflammatory factors, including TNF‐α, IL‐6 and CGRP (Figure 5D–G). These molecules are established critical mediators of migraine pathophysiology, modulating neurovascular crosstalk and nociceptive signaling.

Based on these findings, we propose a mechanistic model wherein endothelial Mt2 serves as a central coordinator of the neuroinflammatory signaling. Specifically, Mt2 appears to integrate endothelial‐derived nitric oxide production (NOS2), cytokine/chemokine release (TNF‐α/IL‐6), and neuropeptide activity (CGRP) to gate vascular tone regulation and neuronal excitability, ultimately driving migraine‐like behavioral manifestations.

## 4 Discussion

**FIGURE 6:**
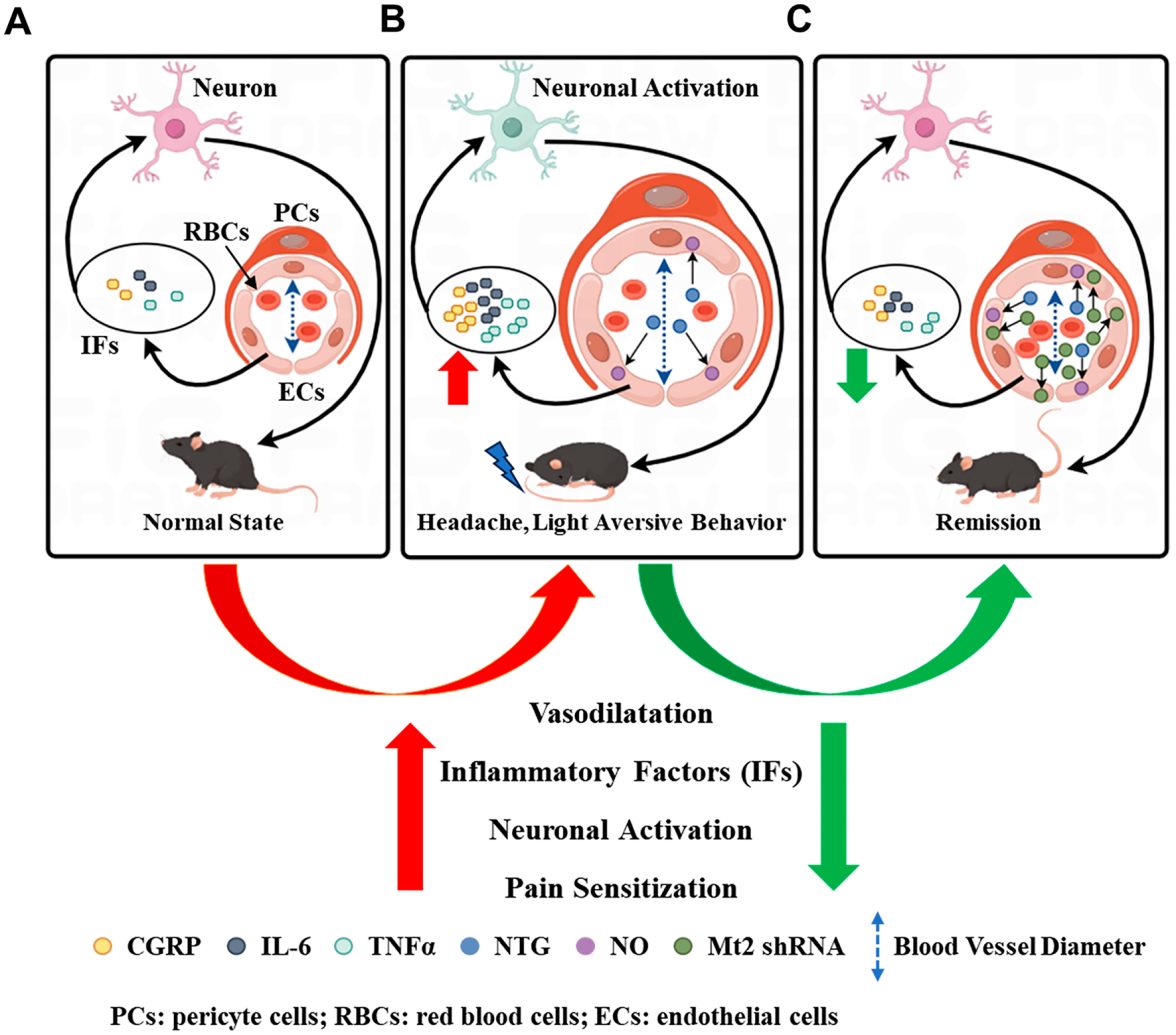
Working model. Wild‐type mice were intraperitoneally injected with nitroglycerin (NTG) to establish a migraine model. NTG promotes nitric oxide (NO) production and induces vasodilation (increased vascular diameter), leading to migraine‐like behaviors in mice—including hyperalgesia (pain sensitivity) and photophobia (light aversive behavior). These behavioral phenotypes are accompanied by intracranial vasodilation, neuronal activation, and elevated release of inflammatory factors (IFs) such as calcitonin gene‐related peptide (CGRP), interleukin‐6 (IL‐6), and tumor necrosis factor‐α (TNF‐α). Notably, knockdown of melanocortin 2 receptor (Mt2) in endothelial cells (ECs) of the somatosensory cortex (SSP) via Mt2 short hairpin RNA (shRNA) significantly reduced the secretion of these pain‐sensitizing mediators and inflammatory factors.

Our study establishes that Mt2 is a critical mediator in CM pathophysiology through its dual regulatory roles in cerebrovascular homeostasis and neuroinflammation. We identified robust Mt2 expression across cerebrovascular endothelial cells and glial populations. The endothelial‐specific knockdown of Mt2 suppressed neurovascular inflammatory cascades (NOS2, TNF‐α, IL‐6, CGRP), regulating the cerebrovascular tone and somatosensory cortical neuronal activation, contributing to the migraine‐like pain relief (Figure 6).

Our previous research has demonstrated that abundant abnormal neuronal activation was found in the somatosensory cortex that is related to migraine‐related headache [24, 25]. In our study, we further identified a marked upregulation of Mt2 expression in the somatosensory cortex, especially in the vascular endothelial cells of NTG‐induced CM mouse models. Given that the somatosensory cortex serves as a critical hub for integrating nociceptive and sensory processing in migraine pathophysiology, this observation prompted us to systematically investigate the pathophysiological role of Mt2 in CM progression.

Emerging evidence indicates that Mt2 exhibits cell type‐ and etiology‐specific roles in neuropathic pain pathogenesis. In spinal cord injury models, Mt2 expression is upregulated specifically in ipsilateral dorsal horn vascular endothelial cells, where it promotes inflammatory mediator release and contributes to pain sensitization. Genetic or pharmacological suppression of Mt2 in this context has been shown to attenuate neuroinflammation and alleviate pain‐related behaviors [11]. However, contrasting findings emerge in oxaliplatin‐induced neuropathic pain models: Mt2 expression is significantly downregulated in sensory neurons rather than endothelial cells, suggesting distinct regulatory mechanisms across pain etiologies. Notably, this regulatory pattern starkly contrasts with observations in oxaliplatin‐induced peripheral neuropathy, where Mt2 expression is markedly downregulated in sensory neurons rather than endothelial cells [11]. These paradoxical findings suggest that Mt2 may engage distinct molecular mechanisms depending on the cell type. In vascular endothelial cells, it likely exacerbates pain through pro‐inflammatory cascades, whereas in neurons, it could exert protective functions by modulating ion homeostasis or mitigating oxidative stress. Collectively, these findings underscore the critical importance of cell type specificity and disease etiology in defining Mt2's roles.

To specifically regulate the expression of Mt2 in the vascular endothelial cell, we generate five‐element nanoparticles (FNPs) developed in which helper polymer poly (β‐amino esters) (PBAEs) and DOTAP are used in combination, with the help of Prof. Li. The new strategy endows FNPs with high stability by increasing the charge repulsion between nanoparticles and the binding force of the aliphatic chains within the nanoparticles. The high efficiency, specificity, and stability delivery platform allows for the specific delivery of Mt2 shRNA to the endothelial cells and is validated by immunofluorescence and immunohistochemistry in Figure 2.

In addition to recurrent episodes of moderate to severe headaches, migraine is also accompanied by associated symptoms including nausea, vomiting, photophobia, and phonophobia. Therefore, in our study, we specifically examined both periorbital mechanical threshold and photophobic phenotypes in an NTG‐induced migraine mouse model. Suppressing the expression of Mt2 in endothelial cells can dramatically relieve periorbital pain and photophobia associated with migraine‐like pain. Therefore, a critical question arises regarding the mechanisms through which vascular endothelial cells regulate migraine‐associated behaviors, particularly given their potential role in modulating neurovascular interactions during migraine pathogenesis.

To further verify this hypothesis, we utilized two‐photon technology to observe vasoconstriction and relaxation functions in the somatosensory cortex. Our experimental data demonstrate that systemic NTG administration induced pronounced cerebrovascular dilation, a hallmark of migraine‐related vascular dysregulation. Notably, targeted genetic knockdown of Mt2 in vascular endothelial cells effectively rescued this pathological vasodilation. This neuronal hyperactivation is considered a pivotal mechanism driving migraine attacks, with modulation of central sensitization demonstrating therapeutic potential in alleviating cephalic allodynia [22]. Thus, NTG induced neuronal activation in the somatosensory cortex, recapitulating migraine neurobiological features. Suppressing vascular endothelial Mt2 reduced this pathological neuronal hyperactivity and ameliorated NTG‐evoked migraine‐like behaviors. Notably, the neuronal function‐regulating CaMKII signaling may be involved, as evidence shows it plays a pivotal role in pain modulation and central sensitization—hallmarks of migraine pathophysiology. For example, in CM, cannabinoid type‐1 receptor activation alleviates central sensitization by inhibiting the NR2B/CaMKII/pCREB pathway, reducing pain mediators like CGRP and SP [23]. CaMKII signaling also contributes to neuropathic pain via regulating neuronal circuits (e.g., PVP^CaMKIIα^ → ZIr^GABA^ → vlPAG^CaMKIIα^) and astrocyte‐associated inflammation, with its inhibition mitigating pain hypersensitivity [24, 25]. Additionally, as a downstream effector of calcium influx (e.g., via TRPV1), it modulates inflammation and cell polarization, linking to pain‐related neuroinflammation [26]. Though CaMKII's direct role in Mt2‐mediated regulation of neuronal hyperactivity remains unclear, these research results indicate that it might be a potential downstream node that can link the inhibitory effect of Mt2 in vascular endothelial cells with the alleviation of neuronal pathological activation in migraines. Further studies are warranted to explore the mechanism of this gene's role in the neurovascular system.

To explore the mechanism that endothelia Mt2 in neurovascular regulation during CM, we performed fluorescence‐activated cell sorting (FACS) of nanoparticle‐labeled vascular endothelial cells followed by transcriptomic profiling. The GO enrichment analysis of DEGs highlighted three interconnected biological themes: vascular endothelial morphogenesis, inflammatory mediator regulation, and vasomotor activity. KEGG pathway analysis further mapped these DEGs to pain‐associated signaling cascades, including PI3K‐Akt, MAPK, and neuroinflammatory NOD/TLR pathways, all previously implicated in peripheral nociception [27, 28, 29, 30]. Experimental validation confirmed Mt2's dual regulatory function: its inhibition reduced NOS2 expression—a key mediator of vascular tone—and concurrently decreased migraine‐related inflammatory markers, including CGRP, IL‐6, and TNF‐α [7, 31, 32]. Notably, as a pivotal factor in trigeminal neuropathic pain, CGRP's roles extend beyond migraine; for example, in trigeminal neuropathic pain models, epigenetic regulation (via ALKBH5‐mediated m6A modification of Htr3a mRNA) modulates pain signaling, underscoring the multifaceted involvement of trigeminal pathways in pain processing [33]. These findings collectively demonstrate that endothelial Mt2 modulates migraine pathophysiology through coordinated regulation of vasomotor dynamics and neuroinflammatory signaling, establishing it as a critical coordinator of neurovascular crosstalk in migraine‐like pain pathway sensitization.

Our study still has many limitations. First, our findings are primarily derived from an NTG‐induced mice model, which reflects acute vascular dilation—a key feature of migraine—but may not fully recapitulate the heterogeneity of human CM pathophysiology, including diverse subtypes, triggers, and neurological manifestations beyond vascular changes [34]. Second, the transcriptomic analyses identified key signaling pathways (like PI3,K‐Akt/MAPK); direct causal links between Mt2 and these pathways require further validation through gain/loss‐of‐function experiments. Lastly, the exclusive use of male mice limits insights into sex‐specific mechanisms, despite the higher prevalence of migraine in females [35]. These limitations highlight the need for multi‐omics human cohort analyses and sex‐balanced preclinical models to fully translate these findings into therapeutic applications.

In conclusion, this study is the first to report that the expression of Mt2 is elevated in CM. We reveal its critical role as a neurovascular modulator through coordinated regulation of endothelial‐mediated vasomotor dynamics (constriction/dilation) and neuroinflammatory signaling. Mechanistically, Mt2 regulates cerebrovascular compliance via modulating the release of migraine‐associated mediators (CGRP, IL‐6, TNF‐α), thereby establishing an interface between vascular tone dysregulation and migraine‐like pain thresholds. Our findings not only delineate Mt2's previously unrecognized function in bridging vascular homeostasis with neural hyperexcitability but also position it as a promising therapeutic target for in CM.

## Author Contributions

C.Z., J. Gao, Y.W., J.L., and X.R. contributed to the design of the work. C.Z., Z.H., K.Z., T.W. contributed to performing the experiment. C.Z. and J. Guan contributed to the acquisition and analysis of data. C.Z. and X.R. drafted the manuscript, and all authors critically reviewed the manuscript before submission.

## Ethics Statement

All experiments with animals were approved by the Animal Ethics Committee (20240827003) of ShanghaiTech University and carried out according to the criteria published by the National Institutes of Health.

## Consent

All authors given consent for the publication.

## Conflicts of Interest

The authors declare no conflicts of interest.

## Supporting information


**Figure S1:** Mt2 exhibits vascular endothelial‐ and microglia‐specific expression patterns.
(A) Representative pictures of Mt2 (red) and CD31 (green) immunofluorescence labeling in the brain. Scale bars =10 μm. (B) Representative pictures of Mt2 (red) and IBA1 (green) immunofluorescence labeling in the brain. Scale bars =10 μm. (C) Representative pictures of Mt2 (red) and NeuN (green) immunofluorescence labeling in the brain. Scale bars =10 μm.
**Figure S2:** Mt2 knockdown attenuates NTG‐induced paw mechanical threshold.
(A) Representative schematic diagrams and procedures for drug administration and behavioral tests. (B, C) Repeated Mt2 shRNA administration decreased paw mechanical hyperalgesia induced by NTG including chronic responses (B) and acute responses (C). Two‐way ANOVA with the Tukey post hoc tests, ##*p* < 0.01, NTG + Mt2 shRNA group compared with NTG + Ctrl shRNA group, *n* = 8/group, **p < 0.01, NTG group compared with Saline group, *n* = 8/group.

**Table S1:** Gene primer sequences used for RT‐PCR.

## Data Availability

The data that supports the findings of this study is available in the Supporting Information of this article. Additional data that support the findings of this study are available from the corresponding author upon reasonable request.
