# Supplementary material for: Endothelial‐Targeted Metallothionein‐2 shRNA Nanoparticles Alleviate Migraine‐Like Symptoms
Source: CNS Neurosci Ther. 2025 Nov 5;31(11):e70643. doi: 10.1111/cns.70643 (PMC12589813; doi:10.1111/cns.70643)
Supplement: Supplementary file 1 — Figure S1: Mt2 exhibits vascular endothelial‐ and microglia‐specific expression patterns. (A) Representative pictures of Mt2 (red) and CD31 (green) immunofluorescence labeling in the brain. Scale bars =10 μm. (B) Representative pictures of Mt2 (red) and IBA1 (green) immunofluorescence labeling in the brain. Scale bars =10 μm. (C) Representative pictures of Mt2 (red) and NeuN (green) immunofluorescence labeling in the brain. Scale bars =10 μm. Figure S2: Mt2 knockdown attenuates NTG‐induced paw mechanical threshold. (A) Representative schematic diagrams and procedures for drug administration and behavioral tests. (B, C) Repeated Mt2 shRNA administration decreased paw mechanical hyperalgesia induced by NTG including chronic responses (B) and acute responses (C). Two‐way ANOVA with the Tukey post hoc tests, ##p < 0.01, NTG + Mt2 shRNA group compared with NTG + Ctrl shRNA group, n = 8/group, **p < 0.01, NTG group compared with Saline group, n = 8/group. [file CNS-31-e70643-s002.pdf]

Fig.S1

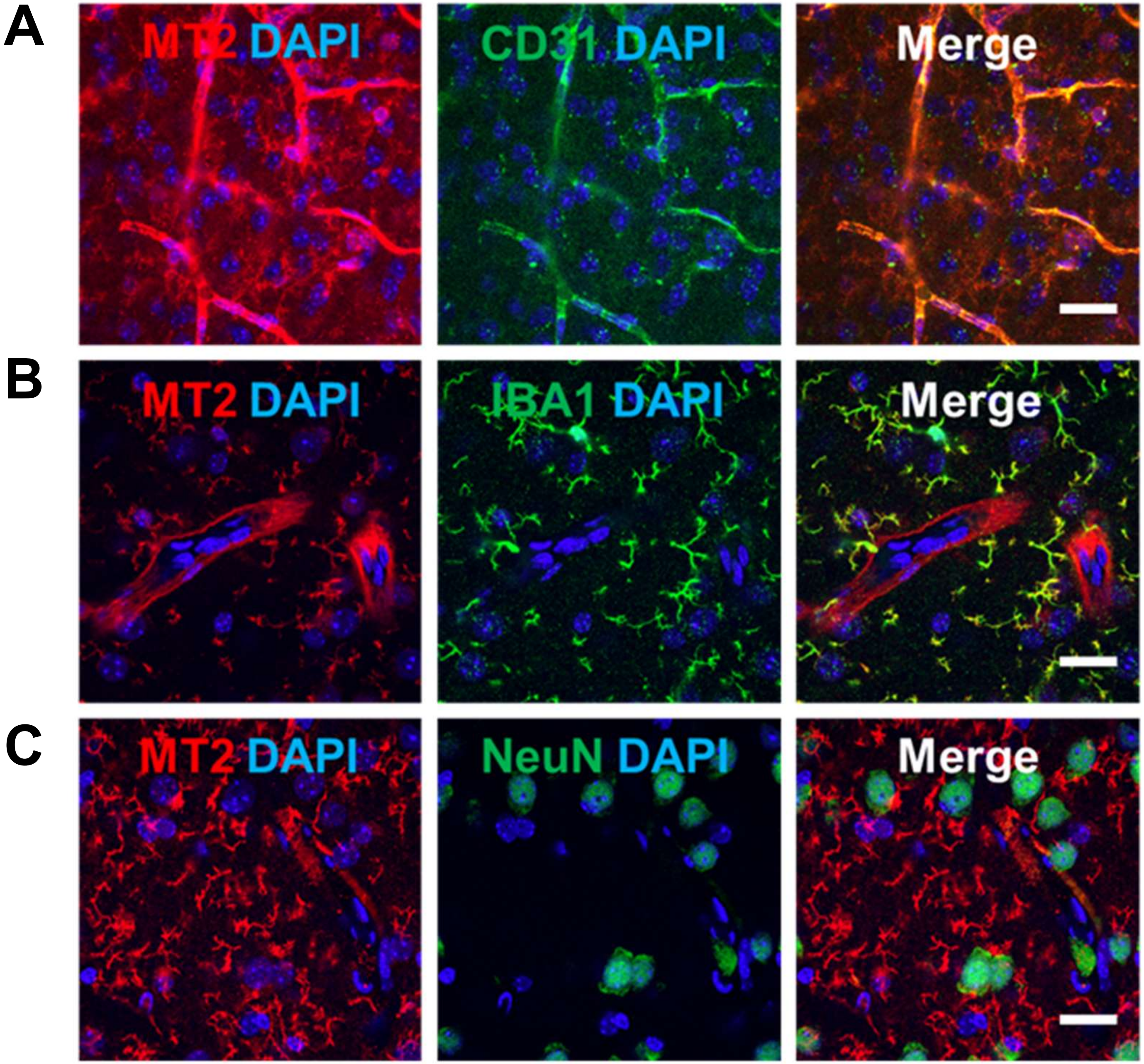
A
MT2 DAPI
CD31 DAPI
Merge
B
MT2 DAPI
IBA1 DAPI
Merge
C
MT2 DAPI
NeuN DAPI
Merge

Fig.S1 Mt2 Exhibits Vascular Endothelial and microglia-Specific Expression Patterns

A. Representative pictures of Mt2 (red) and CD31 (green) immunofluorescence labeling in the brain. Scale bars =10 μm. **B.** Representative pictures of Mt2 (red) and IBA1 (green) immunofluorescence labeling in the brain. Scale bars =10 μm. **C.** Representative pictures of Mt2 (red) and NeuN (green) immunofluorescence labeling in the brain. Scale bars =10 μm.

Fig.S2

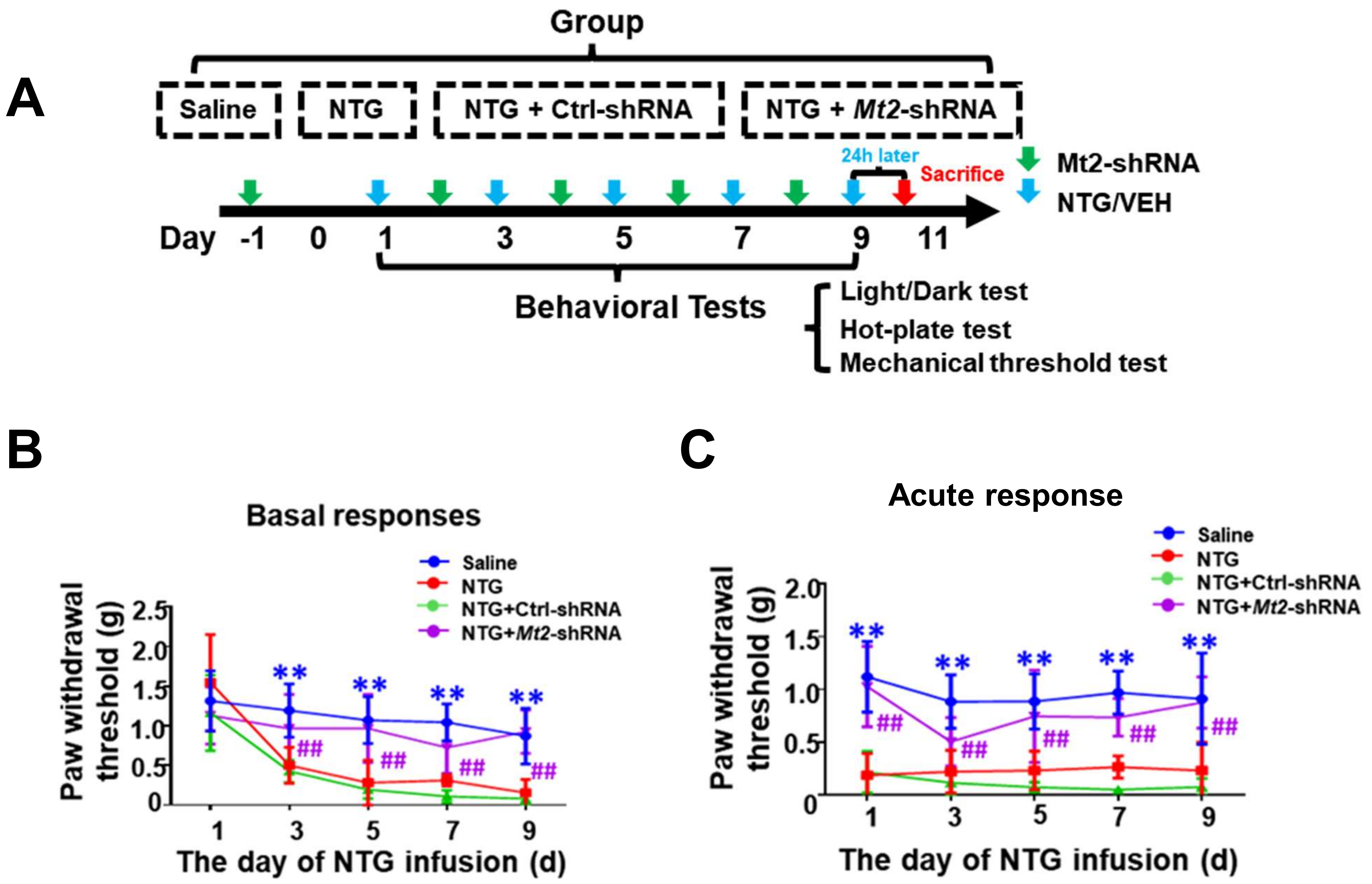
A
Group
Saline
NTG
NTG + Ctrl-shRNA
NTG + Mt2-shRNA
24h later
Sacrifice
Mt2-shRNA
NTG/VEH
Day -1 0 1 3 5 7 9 11
Behavioral Tests
Light/Dark test
Hot-plate test
Mechanical threshold test
B
Basal responses
Saline
NTG
NTG+Ctrl-shRNA
NTG+Mt2-shRNA
Paw withdrawal threshold (g)
The day of NTG infusion (d)
C
Acute response
Saline
NTG
NTG+Ctrl-shRNA
NTG+Mt2-shRNA
Paw withdrawal threshold (g)
The day of NTG infusion (d)

Fig.S2 Mt2 Knockdown Attenuates NTG-Induced paw mechanical threshold

A. Representative schematic diagrams and procedures for drug administration and behavioral tests. **B-C.** Repeated Mt2 shRNA administration decreased paw mechanical hyperalgesia induced by NTG including chronic responses **B** and acute responses **C**. Two-way ANOVA with the Tukey post hoc tests, ##$P < 0.01$, NTG+Mt2 shRNA group compared with NTG+Ctrl shRNA group, $n = 8$/group, **$P < 0.01$, NTG group compared with the Saline group, $n = 8$/group.

.
